# Evaluation of new flexible and integrative psychiatric treatment models in Germany- assessment and preliminary validation of specific program components

**DOI:** 10.1186/s12888-018-1861-1

**Published:** 2018-09-03

**Authors:** Jakob Johne, Sebastian von Peter, Julian Schwarz, Jürgen Timm, Martin Heinze, Yuriy Ignatyev

**Affiliations:** 1 Department of Psychiatry and Psychotherapy, Charité University Medicine Berlin, Charitéplatz 1, 10117 Berlin, Germany; 2 Brandenburg Medical School Theodor Fontane, Department of Psychiatry and Psychotherapy, Immanuel Klinik Rüdersdorf, Rüdersdorf bei Berlin, Germany; 3 Biometry Section, Competence Center for Clinical Trials, University of Bremen, Bremen, Germany

**Keywords:** Flexible, Integrative care, Mental health, Health services research, Regional budget

## Abstract

**Background:**

Flexible and integrative treatment (FIT) models are rather novel in German mental health care. This study aimed at identifying and evaluating empirically based, practicable, and quantifiable program components that describe the specific treatment structures and processes of German FIT models.

**Methods:**

A multi-step, iterative research process, based on Grounded Theory Methodology (GTM), was used to identify and operationalise components. A complex algorithm and expert-interviews were applied to quantify the relative weight of each component and to develop a sum score. Face and content validity were examined and internal consistency was tested by Cronbach’s α coefficient.

**Results:**

Ten of eleven FIT components could be operationalised, quantified and united in the total score. All operationalised components showed sufficient face and content validity and eight components had a good reliability.

**Conclusions:**

The components are a first step in the process of operationally defining German FIT models. They considerably overlap with various critical ingredients of international FIT models and may serve as a theoretical basis for constructing fidelity tools and research guides to enable process and outcome evaluation of German FIT models.

## Background

Flexible and integrative treatment models (shortcut = FIT-models) provided by multi-professional teams (e.g. Assertive Community Treatment (ACT), Home Treatment (HT), Crisis Resolution Teams (CRT) etc.) are widely perceived to be fundamental for adequate mental health care [1]. Accordingly, critical ingredients, evaluation criteria, and fidelity scales have been developed over the past 20 years, among other the Index of Fidelity for ACT (IF-ACT), Dartmouth Assertive Community Treatment Scale (DACTS), Tool for Measurement of ACT (TMACT), Individual Placement and Support Fidelity Scale (IPS25), Core Crisis Resolution Team fidelity scale (Core CRT fidelity scale) [2–8]. Further, a wide array of positive outcomes has been described: reduced costs, lesser rates of hospitalisation, lesser dropout rates, increased housing stability and client and family satisfaction were found to be evident for various community-based treatment models [1, 2, 9].

Despite this evidence, patients with severe mental illness in Germany mostly receive separated in- and outpatient care, with a considerable proportion of inpatient treatment [10]. Further, the German system is characterized by a rather flawed integration of in-patient-services with out-patient and a broad spectrum of rather different psychosocial institutions [10, 11]. Usually, the German system is described to be fragmented [10]. One further striking feature is that outreach services are underdeveloped [12].

**Fig. 1:**
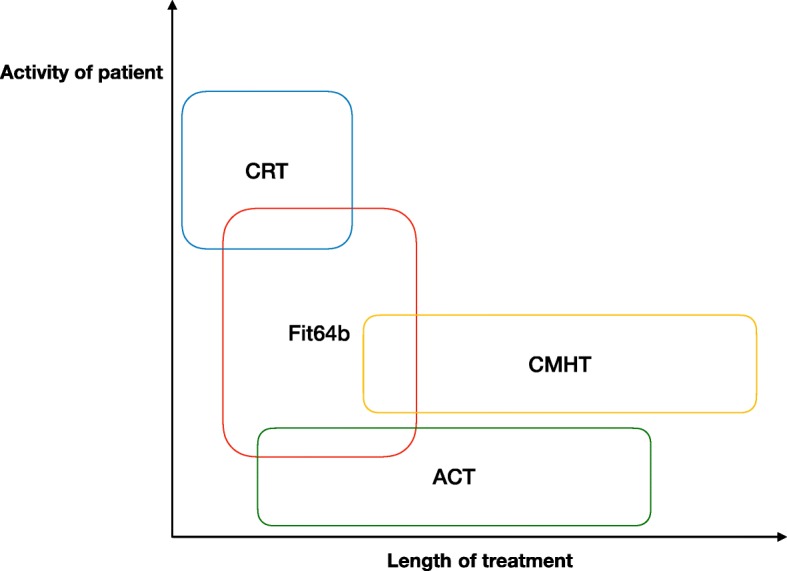
Comparison of FIT models with international models. FIT64b models treat both chronic and acute patients usually for a longer time than CRT models and a shorter duration compared to ACT.

To improve this situation, a law has been introduced recently (year 2013, §64b Book V German Social Law) that allows for new forms of psychiatric flexible and integrative treatment models (shortcut = FIT64b). Contrasting to most international FIT models, and due to local contingencies [13], FIT64b services are offered by hospital-based teams to patients with both acute and chronic conditions (s. Fig. 1). Service providers receive a total budget for all forms of inpatient and hospital-based outpatient care (capitation principle [14, 15]). This budget must cover all expenses, yet, leaving sufficient space for adapting treatments to the needs of a region or patient.

Meanwhile, a total of 20 FIT projects can be found in various parts of Germany. They differ considerably with regard to lengths of services, contextual settings, treatment structures, and processes, depending on historical contingencies and local conditions [16]. At the same time, they all seek to offer continuous, flexible, and integrative forms of care instead of rather brief and rigid sets of mainly inpatient treatment.

Scientific interest and legal directives require careful evaluation of this development. Quantitative data drawn from clinical account data bases are available, but they are not sufficient to evaluate the different FIT processes, outcomes, and evaluation of specific FIT aspects by patients and staff. Moreover, changes following the implementation of FIT models can be difficult to detect, as they are primarily of a budgetary nature. Thus, a theoretical program model had to be developed, delineating program components and integrating the structural and process variations of FIT projects.

The study, EvaMod64b (Evaluation of Models according to SGB §64b), was planned to overcome this gap. The results of the main study will be reported elsewhere (von Peter, Ignatyev et al.: Evaluation of flexible and integrative treatment models in Germany - a mixed method, patient and staff-oriented, explorative study. (in progress)). The aim of this paper was to identify and evaluate an empirically based, practicable, quantifiable, and theoretically sound set of program components that describe the specific treatment structures and processes of German FIT models.

## Methods

### Setting and sampling

This study was approved by the Ethics Committee of Medical Chamber Brandenburg (2016, No. S 7 (a)). All of the 15 hospitals with FIT64b models in 2015 where asked to participate in our study, out of which 12 Departments agreed (Itzehoe, Heide, Rendsburg, Lüneburg, Nordhausen (adult and child/ adolescent psychiatry), Glauchau, Riedstadt (adult and child/adolescent), Berlin-Kreuzberg, Berlin-Neukölln, and Rüdersdorf. The start of FIT64b models varied from January 2013 to January 2016. Seven departments had a prehistory of FIT in the frame work of another social regulation. The examined hospitals are both private (four departments) and public or non-profit (eight departments), and providing care for a regional population of 85,000 up to 425,000 people. Further, some FIT64b models signed contracts with only one or two insurance companies (four departments), other are under contract with all of them (four departments), meaning that in the former, not all patients received the FIT64b specific treatment procedures.

### Assessment of specific program components

**Fig. 2:**
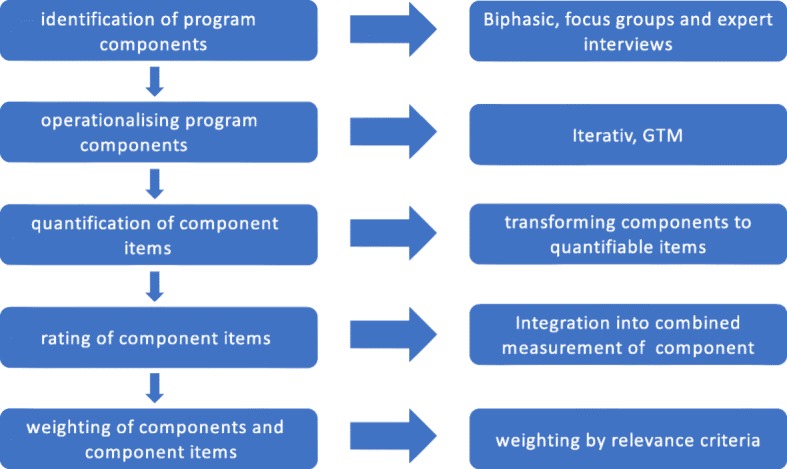
Assessment phases of specific program components

The assessment phase included five steps: 1) identification of program components; 2) operationalising program components; 3) quantification of component items; 4) rating of component items; and 5) weighting of components and component items (s. Fig. 2).

#### Identification of program components

As shown in the introduction, FIT64b models are complex interventions that contain multiple, and interacting, treatment components [17]. Secondly, FIT64b models vary widely, regarding structures and processes. Thirdly, changes following the implementation of FIT64b models can be difficult to detect, as they are primarily of a budgetary nature. Thus, a theoretical program model had to be developed, delineating program components and integrating the structural and process variations of FIT64b projects.

To identify the program components of FIT64b models, data collection was biphasic, occurring in September and October 2015, first in FIT64b projects Departments 1–6, then, after analysing data, in Departments 7–12. Focus groups [18] and expert interviews [19] of staff, patients, and family members were conducted by two authors (SvP, YI) to assess experiences with FIT64b models. The complete research guide cannot be displayed here due to reasons of space, but questions were asked on structural and processual features of FIT64b models, on caused changes in everyday routines and practices, and on perceived benefits and disadvantages.

A total of 14 focus groups and 12 expert interviews across all 9 FIT64b projects were conducted. In total, 24 employees, 16 patients, and four family members were included. Two-thirds of the employees were in management positions, three-quarters were daily workers in FIT64b projects, and three employees worked as controllers. Two-thirds of patients and family members were experienced with standard health care and, thus, were able to compare FIT and conventional psychiatric treatment systems.

Generally, GTM is used for pursuing rule-based and systematic processes of developing explanatory theories [20]. Such kinds of iterative alternations between data collection, analysis, and interpretation were also used to develop the contents of other FIT program components. Data analysis was intermittent. A constant iterative process of data collection and interpretation was performed until data was saturated [21]. Through this process, and over the course of the investigation, a set of recurring FIT64b program components was developed.

#### Operationalising program components

Using the iterative process, according to the GTM, the developed program components were operationalised. For each component, relevant structural and processual criteria were explored and discussed during the above-mentioned expert interviews and focus groups. Component items were constructed that address the main structural and processual changes that have to precede or follow the implementation of FIT64b models.

#### Quantification of component items

The first step of transforming the qualitatively defined and operationalised components to quantifiable items was to quantify each component item with a single value depending on the respective possibilities of answers (0 = not implemented, 1 = partly implemented, 2 = fully implemented). In some cases, percentage ratios of implementation were used. Single items of components I, II, IV, VI, and IX are themselves quantitative as they ask for a count.

Starting quantification was compared with qualitative insights resulting from focus groups and expert interviews. Preliminary quantitative data from a first feasibility data collection in one clinic was integrated. Both resulted in an adjusted quantification using means of some item values (III a, b, c, V a, b and VII a, b, c). A specific problem evolved from the pair a, b, where b is upgrading a. In these cases, they were integrated in one item quantified by the sum. Two items (IX a, b) were eliminated as they seemed to be not specific for the theme of the component.

#### Rating of component items

After development of item quantification, the question arose how to integrate the respective item quantification into a quantification of each component as a whole. The aim of this research step was to integrate the quantified items into a combined measurement of each FIT64b component.

A first attempt to use simple sums of item scores was rejected as the qualitative data showed great differences with respect to the importance between the single items. Instead, ratings of item relevance and relevance weighted sums of items for component, as well as combined components, were introduced.

Rating of the components was done by conducting expert interviews. Eleven academics from examined hospitals who actively dealt with FIT64b were questioned about the importance of the individual component items for quality of life of patients affected by the item. They were asked about the relevance within one year and within three years, and they were asked to state their certainty about the answers given. The answers should be given by scores of one (no impact) to five (strong impact), respectively, and by score one (totally uncertain) to five (totally certain).

#### Weighting of component items and whole components

In order to introduce a weighting of both components and component items by relevance criteria, an algorithm for this weighting process had to be developed. The algorithm, to transform item scores into component scores and a total score of FIT64b compliance, was based on the weighting resulting from expert assessments. In a first step, weighting of item scores affiliated with the component was used to produce component scores for each participating department. In the second step, weights for components were calculated in order to create a total score measuring the FIT64b compliance of the departments in general. In a third step, these component weights were applied to the department component scores produced in step one.

The algorithm used was developed as follows: Let Fij be the score given by clinic j assessing the realisation of item i. The weighted sum of items for a component k and clinic j may be written as Mjk = sum(pik * Fij), where sum is calculated over all items i contained in component k and sum(pik) = 1 for each k. The factors, pik, are derived from the expert votes as follows: let Ai be the mean of expert relevance ratings for item i (mean over all expert ratings for one year as well as three years). Let S1i and S3i be the mean scores of certainty assessment over all experts. As this measurement of uncertainty is subjective, we combined it with a more objective score D1i, respectively, D3i defined by the inverse standard deviation of expert assessments for item i. By this we got combined certainty assessments C1i = f * S1i + D1i, C3i = f * S1i + D1i, and Ci = mean(C1i,C3i). The factor f is used to balance the terms in the sum. We used f = 2.5. The product of certainty and mean assessment gi = Ai * Ci, yields the wanted weighting factor pik = gi / Gk, where Gk = sum(all gi belonging to k). In order to get an integrated measure for FIT compliance, we calculated a weighted sum of the resulting component values, Mjk, for clinic k. This is simply done by using the same weight factors as above, i.e. MTj = sum(pk * Mjk), where k = 1,..,11, and pk = Gk / G with G = sum(Gk). Greater importance was given to components with weight of 10% or more regarding to total score.

### Testing internal consistency and content validity

The validity phase included examining the face and content validity of FIT64b program components, as well as their internal consistence.

To test face validity, all program components were continuously discussed with staff and patients of FIT64b models during data collection. Further, a steering committee was established, consisting of the research team and of FIT64b project staffs, spanning all occupational groups, as well as of two representatives of patients and family members. This committee critically discussed and re-evaluated the components’ validity and practicability before initiating the main study.

To examine the content validity of program components, the content validity ratio CVR [22] was calculated for each item based on the expert interviews with mean CVRs for each component and overall mean. The formula CVR = (Ne-N2)/N2 was used on the item level where Ne is the number of experts votes “essential” while N is the number of experts and N2 = N/2. In a first step the raw items presented to the experts were examined in this way. As the scores ranged from 1 to 5 (no impact to strong impact) a score of at least 3 was interpreted as essential in the sense of this CVR analysis. In a second step the effect of our weighting approach was checked by the same procedure for the weighted items, Gi = Si*Ai (see above), calculated separately for each expert and item. The Gi where defined to indicate essential scores if they were greater than the value, resulting by the same border of 3 for assessments and individual certainty together with a standard deviation smaller than that of an equal distribution.

To test the internal consistency of components, the estimation of Cronbach’s α internal reliability coefficient was performed. The testing of reliability was carried out on the item component as well as component’s weighted sum score levels. In a first step, the reliability of the base for the weighting algorithm was evaluated by testing agreement between experts assessing the relevance of each item of the components and their own estimate about the certainty of this assessment for a one year and a three-year time interval. A total of four sets (assessments and certainty for one and three years), regarding the 33 component items assessed by 10 experts, was tested by Cronbach’s alpha [23].

In a second step, the reliability of the whole instrument, i.e. the weighting algorithm, was evaluated by constructing new weights by the same formal algorithm, not using the mean of the expert votes but applying the algorithm in a different way for each expert separately based on their individual assessments. This procedure yielded 10 different FIT64b measurements on the component level (10 components), as well as on the level of weighted sum of components, which were applied to the answers of the 12 departments involved in the study as a test data set. A Cronbach’s α between 0.6 and 0.7 is considered an acceptable value. A value between 0.7 and 0.9 is a good value, and a value of 0.9 or higher indicates excellent reliability [24].

## Results

### Program components

The complex, multi-step, and iterative research process based on GTM resulted into 11 components of FIT64b programs, addressing the main areas of change from conventional to FIT64b oriented psychiatric treatment.

For reasons of space, each step of development on the basis of citations cannot be displayed here. Open coding in the Departments 1–6 resulted into program components I, II, IV, V, VI, and VII (see Table 1).

**Table 1:** Operationalization of FIT components

No.	Component	Operationalisation	Assessment
I	Shifting in- to outpatient setting *Shift of treatment from I* ^*1*^ *towards D* ^*2*^ *and/or O* ^*3*^	• Number of outpatient SoF^4^/total number SoF^4^ during EP^5^	
II	Flexible care management across settings *Unproblematic shift of SoF* ^*4*^ *(prompt, little bureaucracy*	• Number of CoT^6^ using all three SoF^4^ during EP^5^/ total number CoT^6^ • Treatment D^2^, I^1^, and/or O^3^ in the same unit (ward, level etc.) • Systematic steering of treatment beyond all SoFs^4^ • Application of SoF^4^ spanning roster and therapy plans	Rating scale (0–2)
		• Number SoF^4^-spanning sessions (meetings etc.)	Rating scale (1–3)
III	Continuity of treatment team *Implementation of team- and individual-related continuity*	• Percentage of staff working in more than one SoF^4^ (on a regular basis) • Coordinated admission (coordinating staff member) • Coordination of treatment by e.g. case manager, SoF^4^-spanning care • Home treatment by I^1^- and D^2^- teams • Outsourced PIA (outpatient department) team (not working in I^1^ or D^2^)	Rating scale (0–2)
IV	Multiprofessional Cooperation *Intense multiprofessional cooperation*	• Absolute number of mandatory sessions across all occupational groups	Absolute number
		• Measure/action to optimize cooperation across all occupational groups	Rating scale (0–1)
		• Training sessions multiprofessional cooperation	
		• Number occupational groups working in home treatment (on a regular basis)	Rating scale (0–2)
V	Therapeutic group sessions across all settings *Therapeutic groups with members from all SoF* ^*4*^	• Number of group sessions open for all SoFs^4^	Rating scale (0–2)
VI	Outreach home care *Multiprofessional treatment at home ≥ 1 week*	• Number CoT^6^ with home-treatment/ all I^1^-cases during EP^5^	
		• Cars for home-visits	Rating scale (0–2)
VII	Involvement of informal caregivers *Informal caregivers as therapeutic tool*	• “Network” or other forms of systemic dialog with informal caregivers and/or “carer-conference” and/or “caregiver groups”	Rating scale (0–1)
		• Number of groups open for informal caregivers	Rating scale (0–1)
		• Percentage of systemic training for staff/employees (e.g. open dialogue)	Percentage
VIII	Accessibility of services *Geographical accessibility and accessibility of teams*	• Accessibility of services within one-hour drive • 24-h-accessibility of multiprofessional mental health team (not doctor on call or the like) • Shuttle service for services users	Rating scale (0–2)
		• Waiting list	Reverse rating scale (1–0)
IX	Sovereign steering of services *Freedom of therapeutic decisions*	• Number of exeats ≥2 nights in a row/all exeats during EP • Number of exeats per service user/ calendar week during EP • Daypatient treatment as well during the night • Rules according to contract in all matters concerning setting of treatment and length of treatment	Rating scale (0–2)
X	Cooperation across Sectors *Cooperation with ambulant care systems*	• Mutual scheduling and realizing of treatment with ambulant care systems (SGB V) • Mutual scheduling and realizing of treatment with social welfare system (SGB XII)	Rating scale (0–2)
		• “Community psychiatric network”	Rating scale (0–1)
XI	Expansion of professional expertise *Professionalisation of staff*	• Multiprofessional training of staff concerning FIT models • Measures to multiply knowledge about FIT models • FIT models as part of appraisal interviews	Rating scale (0–1)
		• Percentage of nurses/caregivers moderating group sessions	Percentage

NOTE: ^1^*I* inpatient, ^2^*D* day-patient, ^3^*O* outpatient, ^4^*SoF* setting of treatment (outpatient, day-patient, inpatient), ^5^*EP* evaluation period, ^6^*CoT* case of treatment

Following data collection in Departments 1–6, data were analysed with respect to these six initial components. They were further validated during the second phase of data collection in Departments 7–12. In addition, four components (III, VIII, IX, and XI, see Table 1) were added.

Data from both phases of data collection were analysed. When presented to the steering committee, the specific value of component V and IX were extensively debated, but finally accepted; as both components occur widely in non-FIT64b models. An additional component was suggested, component X, as FIT64b models in Germany also attempt to transcend hospital and other care models, and due to the fact that this component has also been described to be critical for flexible and integrative care models.

### Operationalisation

Operationalised items are shown in Table 1. Due to reasons of space, only the most important changes during their course of development, and following their continuous validation during data collection and evaluation within the steering committee, are described here: Operationalisation of component I and partially of component VI was questioned, as it was unclear if it was a mediator or outcome itself. A patient-related operationalisation for component II could not be found; thus, the current ones refer to institutional changes. The remoteness of outpatient clinics largely prevents flexibilisation of treatments and, thus, was included in operationalising component III. The split between both the space- and team-related operationalisations of component VIII created a lack of unity during analyses. And finally, component II and IX were conceptually strongly related, thus mainly differing due to their calibration.

### Quantification

Item answer scores, quantifying the operationalised components, were collected from the 12 study departments (Table 2). The values of these scores ranged from 0 to 12 with a mean of 1.17 and standard deviation of 1.55. Data for component I and in part for component VI were not included (see component I and VI problems in section “Operationalisation”). In some cases (component III, V, and VII), answers (3, 2 and 3 answers respectively) were combined to one item score. The only item of component VI was scored by “1” because of lack of variation in all departments.

**Table 2:** Quantification of components by items collected from 12 departments

Component	Item characteristic			
	Mean	SD^1^	Min^2^	Max^2^
II	2.31	1.19	0	4
III	0.62	0.71	0	2
IV	2.44	3.31	0	12
V	2.13	0.93	1	4
VI	1.00	0.00	1	1
VII	0.55	0.58	0	2
VIII	0.65	0.48	0	1
IX	0.67	0.48	0	1
X	0.61	0.55	0	2
XI	0.88	0.53	0	2
Total	1.17	1.55	0	12

Note: ^1^ *SD* standard deviation, ^2^*Min; Max* minimal and maximal value

### Rating

Return rate of questionnaires was high (90.9%). Experts expected increasing effects of FIT64b models on the long run with an average expectation of 3.64 for one year and 3.89 for three years. Small variations in ratings could be detected with a standard deviation of 0.398 and 0.382 (Table 3).

**Table 3:** Psychometric properties and relative weights of program FIT components

		Program component										
Measure		C2	C3	C4	C5	C6	C7	C8	C9	C10	C11	Total score
Number of related items		4	5	4	1	1	3	4	4	3	4	33
Item relevance within 1 year	M (SD)^1^	3.38 (1.21)	3.60 (1.18)	3.40 (1.03)	3.60 (1.17)	4.10 (0.57)	3.70 (0.99)	3.78 (1.00)	4.05 (0.99)	3.50 (0.73)	3.55 (0.90)	3.63 (1.03)
	Min; Max^2^	1;5	1;5	2;5	2;5	3;5	2;5	2;5	2;5	2;5	2;5	1;5
Certainty about this assessment	M (SD)	3.65 (0.89)	3.68 (0.91)	3.70 (0.95)	4.00 (0.47)	3.63 (1.27)	3.63 (1.27)	3.63 (1.00)	3.83 (1.13)	3.77 (0.63)	3.85 (1.21)	3.47 (0.97)
	Min; Max	2;5	2;5	2;5	2;5	3;5	1;5	2;5	1;5	2;5	1;5	1;5
Item relevance within 3 year	M (SD)	3.77 (1.06)	3.90 (0.86)	3.73 (0.99)	3.90 (0.88)	4.50 (0.53)	4.03 (0.93)	3.85 (1.00)	4.15 (0.92)	3.77 (0.57)	3.65 (0.86)	3.87 (0.92)
	Min; Max	1;5	2;5	2;5	3;5	4;5	2;5	2;5	2;5	2;5	2;5	1;5
Certainty about this assessment	M (SD)	3.63 (0.90)	3.62 (0.95)	3.63 (0.87)	3.60 (1.17)	3.80 (0.79)	3.50 (1.41)	3.50 (1.06)	3.65 (1.27)	3.60 (0.97)	3.55 (0.99)	3.59 (1.04)
	Min; Max	2;5	2;5	1;5	1;5	2;5	1;5	1;5	1;5	1;5	1;5	1;5
Content validity ratio (CVR)		0.65	0.88	0.80	0.99	0.99	0.73	0.75	0.80	0.99	0.95	0.87
Cronbach’s alpha		0.84	0.82	0.81	n.a.^3^	n.a.	0.85	0.83	0.85	0.85	0.81	0.93
Relative weight of each component (%)		11.21	16.03	12.20	2.99	4.73	10.63	12.10	6.35	10.87	12.89	100

Note: ^1^*M* mean, *SD* standard deviation, ^2^ *Min; Max* minimal and maximal value, ^3^*n.a.* not availabe

### Weighting

Whereas components II, III, IV, VII, VIII, X, and XI achieved relative weight of 10% or more regarding to total score, components V, VI, and IX were of smaller importance (Table 3.)

**Fig. 3:**
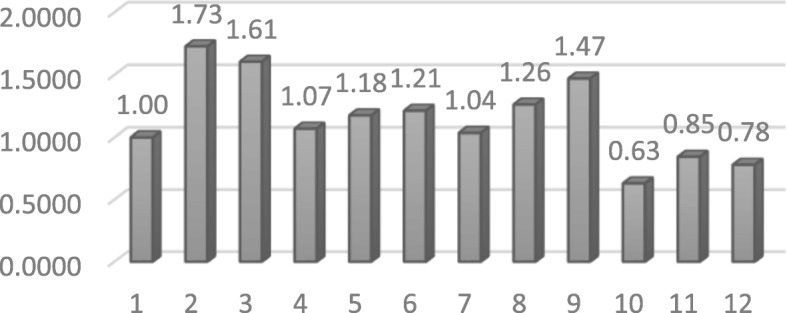
Total score (weighted sum of component scores) for each of twelve participating department

The resulting total scores for each department are presented in Fig. 3 as an example of this measurement, illustrating the different FIT64b status of the involved departments. The values range from 0.63 to 1.73, with mean of 1.15 and standard deviation of 0.33.

### Validity and reliability

The Grounded Theory approach required extensive face validation during the process of developing FIT64b specific components. Further, when presented to the steering committee, all components were found to be sufficiently practicable and extensive to describe FIT64b models.

When ten experts were interviewed, Lawshe’s recommended cut-off content validity ratio of 0,62 was reached for each program component (Table 3).

The resulting Cronbach’s alphas (Table 3) document a good reliability of eight program components and an excellent reliability for the total score.

The internal consistency of components I, V, and VI could not be evaluated, because of missing data for component I and only one item (i.e. no weighting) for components V and VI.

## Discussion

### Main findings

The declared objective of the recently implemented FIT64b models in Germany is the continuous resolution of highly institutionalised inpatient care, as had already been achieved in various health care systems worldwide by the implementation of community based, flexible, and integrative care models [25–29]. The German health care system entails its own legal and organisational logics [30]. Thus, internationally existing research on critical components, or fidelity and evaluative criteria of flexible and integrative forms of psychiatric care (FIT models), does not reflect the local situation. Consequently, 11 FIT64b program components were developed for describing the specific treatment structures and processes of German FIT64b models.

The identified FIT64b specific program components were: I transfer to outpatient setting; II flexible shift of settings; III continuity of treatment team; IV cooperation across all occupational groups; V therapeutic group sessions across all settings; VI outreach care; VII systematic inclusion of informal caregivers; VIII accessibility of services; IX sovereign steering of therapeutic decisions; X cooperation across sectors; XI expansion of professional expertise. Ten of eleven FIT components could be operationalised, quantified and united in the total score. All operationalised components showed sufficient content validity and eight components had a good reliability. The highest weights in the total FIT64b score had components II, III, IV, VII, VIII, IX, X, XI. However, components V and VI could be operationalised by only one feature and had relatively low weights in the total FIT64b score.

### Strength and limitations

This is the first English-language publication describing specific components of FIT models applicable to the German mental health care system. In several studies, focus groups and interviews were used to develop critical ingredients and evaluative criteria of international FIT models [4, 8, 25, 31–33]. Most of them, however, used data from only service users or practitioners [25, 31]. To our knowledge, only two publications collected data from all three stakeholder groups, i.e. from service users, carers, and practitioners [8, 33]. Yet, in our study, the response rates by carers were rather low, maybe due to the fact that carers are not sufficiently involved within FIT64b treatment models (von Peter, Ignatyev et al.: Evaluation of flexible and integrative treatment models in Germany - a mixed method, patient and staff-oriented, explorative study. (in progress)).

The identifying of program components was carried out using a GTM approach that enabled to combine an empirically based, open proceeding with a systematic and rule-based theory construction [20]. In contrast to methods, such as concept mapping or thematic analysis that are frequently used to develop evaluative or fidelity criteria e.g. [8], the method of GTM allows to develop a primarily praxis-based, both empirically and theoretically saturated, middle range theory by gradually building categories and relating them to each other [34]. A continued processing of the components is planed during a further study (von Peter, Ignatyev et al.: Evaluation of flexible and integrative treatment models in Germany - a mixed method, patient and staff-oriented, explorative study. (in progress)), aiming at differentiating them into core and peripheral ones [3, 25, 35].

Further, our findings are limited by the unavailability of a standardised instrument for assessing and measuring FIT64b components. The component “transfer to outpatient setting” could not be operationalised, and quantified because it was unclear if this component was a mediator or outcome itself. Testing internal consistency and content validity of all components were performed only using expert survey for pragmatic reasons. The expert panel was composed from academics of examined departments, which can lead to overestimating FIT64b components and be subject to biases. Furthermore, the present sample included only 12 mental health care departments which may limit the generalizability to other German FIT models.

### Comparison with the literature

The 11 identified program components mostly comply with critical ingredients of international FIT models [4, 25, 32, 36, 37], as they address the transformation processes from conventionally institutionalised towards flexible and integrated forms of psychiatric care. Critical ingredients of flexible and integrative care models vary due to local specifics of national health services. Involved within these variations are various factors, such as availability of resources, stage of program development, geographic location, and populations served [25]. Thus, compared internationally, our study revealed several similarities or distinctions concerning critical components of FIT models.

The key role of components “continuity of treatment team” and “flexible care management across settings” in German FIT64b is comparable with international FIT models [2]. These treatment approaches have a relatively long tradition in Germany as they are useful coordinating mechanisms in the situation of the highly fragmented national health care provision [38, 39].

The developing component “cooperation across sectors” can be also explained as a reaction to the decentralized, multi-layered German health care system [40]. Yet, in the attempt to empirically ground the latter component during our study, data achieved low saturation. This can be explained by the fact the underlying §64b legislation primary yields at integration of various forms of hospital care, but not beyond.

Component VI (outreach home care) had a relatively low statistical importance in our sample compared to other program components. This reflects that outreach care in Germany is little developed. In contrast, various models of assertive and outreach treatment are integral parts of several international FIT models; overlapping criteria can particularly be found for CRT and ACT treatment models, and, for example in the US the development of home treatment was political motivated as a reaction to the anti-psychiatric movement and two waves of deinstitutionalization [41]. On the other hand, all experts agreed that this component should be strongly developed in the future through the FIT64b-programmes and that its availability represents a good indicator for the quality of FIT-programs.

The developing components “inclusion of informal caregivers” and “accessibility of services” could be compensatory mechanisms to close the gap [40]. They are consistent with existent literature [8, 25, 31–33]. The components “multiprofessional cooperation” and “expansion of professional expertise” are in the actual trend with modern German medical education system [42].

## Conclusions

The described program components are a first step in the process of operationally defining German FIT64b models.

Regarding scientific interests, the components allow for an integrated process of collecting, analysing, interpreting, and representing both qualitative and quantitative sets of data. They, thus, may help to overcome widely acknowledged challenges of mixed method designs in health services research [43, 44]. By this way, they may serve as a theoretical basis for constructing fidelity tools and research guides to enable process and outcome evaluation of German FIT models.

Further, the specific FIT components identified may be useful for the implementation of German FIT64b models. They can serve as a target figure for the implementation and/or monitoring of FIT64b projects. By helping to bear in mind the different treatment components, they pave the way for purposive strategies of establishing or developing German FIT models.

## Abbreviations

ACT: Assertive Community Treatment
Core CRT fidelity scale: Core Crisis Resolution Team fidelity scale
CRT: Crisis Resolution Teams
DACTS: Dartmouth Assertive Community Treatment Scale
EvaMod64b: Evaluation of Models according to SGB §64b
FIT: Flexible and integrative treatment models
HT: Home Treatment
IF-ACT: Index of Fidelity for ACT
IPS25: Individual Placement and Support Fidelity Scale
TMACT: Tool for Measurement of ACT
